# Increased Trophoblast Cell Ferroptosis via HMGB1/ACSL4 Pathway Is Associated with Spontaneous Abortion

**DOI:** 10.1007/s43032-025-01817-7

**Published:** 2025-02-24

**Authors:** Yishan Dong, Yong Li, Wenjie Tang, Qin Chen, Chengcai Kong

**Affiliations:** 1 Department of Gynecology and Obstetrics, Changzhou Maternity and Child Health Care Hospital, Changzhou Medical Center, Nanjing Medical University, Changzhou, China; 2 Department of Gynecology and Obstetrics, Changzhou Maternity and Child Health Care Hospital, Changzhou, China; 3 Department of Gynecology and Obstetrics, Maoming Maternity and Child Health Care Hospital, Maoming, China

**Keywords:** HMGB1/ACSL4 pathway, Ferroptosis, Spontaneous abortion, Trophoblast cell

## Abstract

**Introduction:**

Trophoblast cells undergo ferroptosis in pregnancy-related diseases. HMGB1 participates in pathological ferroptosis. However, whether lipopolysaccharide (LPS) -mediated HMGB1 expression induces the ferroptosis of trophoblast cells and further spontaneous abortion (SA) remains unknown.

**Methods:**

HMGB1 and ACSL4 expression were measured in villous tissues from 20 women with SA and 20 women with elective abortion. Human HTR-8/SVneo cells were treated with LPS to establish an in vitro abortion model. The hallmarks of ferroptosis including MDA, GSH, Fe^2+^ and ROS were detected using indicated assay kits.

**Results:**

The levels of HMGB1 and ACSL4 in villous tissues from SA women were significantly higher than those in the normal control group. HMGB1 interacts with and stabilizes ACSL4 to promote the ferroptosis of trophoblast cells. Conversely, HMGB1 and/or ACSL4 inhibition attenuated LPS-induced trophoblast cells ferroptosis.

**Conclusions:**

An HMGB1/ACSL4 axis is engaged in LPS-induced ferroptosis of trophoblast cells, and may be targeted to design treatments preventing SA.

**Supplementary Information:**

The online version contains supplementary material available at 10.1007/s43032-025-01817-7.

## Introduction

Spontaneous abortion (SA), defined as the spontaneous loss of a pregnancy before the embryo or fetus reaches viable, is the most common adverse event of pregnancy [1, 2]. Major causes of SA involve fetal chromosomal abnormalities, infectious inflammation, uterine structural defects, maternal immune abnormalities, and endocrine problems [3]. However, SA of unknown reason is also frequent, necessitating deeper exploration for underlying molecular mechanisms.

Ferroptosis, a lipid peroxidation-induced programmed cell death, develops with the accumulation of free irons, reactive oxygen species (ROS), and lipid peroxidation. Besides, disruption of the antioxidant pathway of SLC7A11/GSH/GPX4 promotes lipid peroxide accumulation and increases ROS levels within cells, thereby contributing to ferroptosis. Recently studies have shown that ferroptosis plays critical roles in cancers and neuroinflammation. Fascin activates ferroptosis in breast cancer by increasing xCT degradation [4]. G9a induces neuronal vulnerability to inflammatory stress through transcriptional control of ferroptosis [5]. Ferroptosis is also involved in pregnancy-related diseases, like SA, gestational diabetes mellitus, and pre-eclampsia [6–8]. Hypoxia induces the ferroptosis of trophoblast cells by activating the lnc-HZ06/HIF1a-SUMO/NCOA4 axis, probably resulting in miscarriage [9], and inhibition of ferroptosis by Shoutai Pill (a traditional Chinese medicine prescription) exerts a preventive effect on pregnancy loss [10].

High-mobility group box 1 (HMGB1), a highly conserved nonhistone nuclear protein, belongs to a family of damage associated molecular patterns (DAMPs) [11]. HMGB1 participates in the progression and deterioration of many diseases, including cancers, trauma, and autoimmune diseases [12, 13]. Once released into the extracellular, HMGB1 active the HMGB1/NF-κB pathway, HMGB1/RAGE pathway, and HMGB1/JAK/STAT pathway to arouse inflammatory responses [14]. Recent studies have demonstrated the implication of HMGB1 in pregnancy processes. Excessive HMGB1 in intrauterine cavity or maternal circulation leads to SA and premature delivery [15]. Feng Zhou et al. have further shown that HMGB1 participates in LPS-induced in autophagy of trophoblasts, leading to inflammatory responses or even miscarriage [1]. In acute kidney injury, cytoplasmic HMGB1 induces ferroptosis and cell death via ACSL4 [16]. ACSL4 is involved in the biosynthesis and catabolism of fatty acids, serving as a biomarker of ferroptosis. However, how HMGB1 and ACSL4 co-regulate the ferroptosis in SA has not been elucidated.

In this study, we hypothesized that HMGB1 and ACSL4 participate in villous injury via ferroptosis. We noted that expression levels of HMGB1 and ACSL4 were markedly increased in trophoblasts ferroptosis. Furthermore, we developed an in vitro model of SA utilizing LPS-stimulated HTR-8/SVneo cells. An HMGB1/ACSL4 axis is suggested to regulate trophoblasts ferroptosis, thus providing a new insight into the pathogenesis and treatment of SA.

## Materials and Methods

### Patients and Sample Collection

All samples were obtained from Changzhou Maternity and Child Health Care Hospital between January 2023 and June 2023. Placental villous samples were collected from 20 women who experienced SA at 6–12 weeks. Women with immune disorders, endocrine imbalances, genetic diseases, genital malformation, infection and other factors were excluded. Tissues in the normal control (NA) group were obtained from 20 age-matched women who underwent elective abortion. Women in the NA group had no history of diabetes, hypertension or adverse pregnancy. Both the SA and NA groups underwent vacuum aspiration to collect intrauterine tissue containing villi, which was then washed by PBS, stored at -80℃ or fixed in formalin for further study. The present study was approved by the Ethics Committee of Changzhou Maternity and Child Health Care Hospital. All patients signed informed consent before sample collection.

### Immunohistochemical Staining

Tissue specimens were fixed in formalin immediately, followed by standard paraffin embedding. The sections were boiled in citrate buffer (pH 6.0) for 10 min for antigen retrieval. Hydrogen peroxide (3%) in methanol was used to eliminate endogenous peroxidase activity. Then the sections were blocked using 5% BSA (Beijing Solarbio Science & Technology Co., Ltd), labeled at 4℃ with anti-HMGB1 antibody (1:100, Proteintech) or anti-ACSL4 antibody (1:100, Proteintech) overnight, then biotinylated with horseradish peroxidase (HRP)-conjugated rabbit secondary antibody at room temperature for 1 h. The sections were placed in the solution for DAB reaction and examined with a microscope.

### Western Blot

Total protein was extracted from villous tissues or HTR-8/SVneo cells with RIPA lysis buffer (Beyotime, China) containing protease inhibitors (Beyotime, China). Every 20 µg of protein was separated from each sample by 10% SDS-PAGE, transferred to PVDF membranes and blocked with 5% skimmed milk in TBST for 1 h at room temperature. The membranes were incubated with specific primary antibodies, including anti-HMGB1 (1:1000, Proteintech), anti-ACSL4 (1:1000, Proteintech) and anti-β-actin (1:5000, Bioworld) overnight at 4℃. Then, the PVDF membranes were incubated with HRP-conjugated secondary antibodies for 1 h at room temperature, and visualized by chemiluminescence. The intensity of each band was quantified by Image J.

### Cell Culture

Human trophoblast cell line HTR-8/SVneo was purchased from Shanghai Zhong Qiao Xin Zhou Biotechnology Co., Ltd., cultured in RPMI-1640 (Gibco) containing 10% FBS (Gibco) and 1% penicillin/streptomycin (Gibco BRL, Grand Island, NY, USA) at 37℃ in a 5% CO_2_ incubator. When the confluence reached 80% or above, the cells were treated with 100 ng/ml LPS (Sigma, St. Louis, MO, USA) for 24 h.

### Cell Viability

Cell viability was measured using Cell Counting Kit-8 (CCK-8, PF00004, Proteintech Group, Inc). HTR-8/SVneo cells were seeded into 96-well plates at a density of 5000 cells/well and treated as indicated for 24 h. Then, 10 µl of incubation reagent and 90 µl of medium were added to each well and incubated at 37 ℃ for 1 h. Absorbance was measured at 450 nm by a microplate reader (BioTek Instruments, Inc.).

### Malondialdehyde (MDA) Assessment

The relative concentration of lipid peroxidation was calculated by MDA level measured using an MDA assay kit (Beyotime, S0131S) according to the manufacturer’s instructions. The MDA in each sample was reacted with thiobarbituric acid (TBA) to form an MDA-TBA mixture that was then measured colorimetrically at 532 nm using a microplate reader (BioTek Instruments, Inc.).

### Glutathione (GSH) Measurement

The relative GSH level was measured by a GSH assay kit (Beyotime, S0053) according to the manufacturer’s instructions. Briefly, the sample was mixed with the GSH working solution, and the absorbance was read at 405 nm by a microplate reader (BioTek Instruments).

### Iron Assay

The relative ferrous iron (Fe^2+^) level was tested using an iron assay kit (Abcam, ab83366, Cambridge, UK) according to the manufacturer’s manuals. Briefly, 5 µl of iron reducer was added to each sample and the reaction was incubated at 25℃ for 30 min. Then, 100 µl of the iron probe was added to the mixture for 60 min in the dark. Finally, the absorbance was measured at 593 nm.

### Reactive Oxygen Species (ROS) Measurement

The intracellular level of ROS was assessed using a cellular ROS assay (Beyotime, S0033S). The cell culture medium was removed and incubated with 10 µM DCFH-DA at 37℃ for 20 min. After removing excessive DCFH-DA by washing thrice with PBS, the cells were visualized under a fluorescence microscope (Leica).

### Cell Transfections

Overexpression plasmids pCMV3-HMGB1-Flag and pCMV3-HA-ACSL4 were bought from SinoBiological (Beijing, China). siRNAs targeting HMGB1 or ACSL4 (RiboBio, Guangzhou, China) were used to knock down the HMGB1 or ACSL4 gene in HTR-8/SVneo cells. The HMGB1 and ACSL4 overexpression plasmids were transiently transfected by Lipofectamine 3000 (Invitrogen). si-HMGB1 and si-ACSL4 were transfected using riboFECT™ CP (RiboBio) according to the manufacturer’s instructions.

### CHX Chase Assay

After transfection with plasmid pCMV3-HMGB1-Flag and the vector control for 48 h, HTR-8/SVneo cells were treated with CHX (50µM) for 0, 1.5, 3, and 6 h, respectively. Total proteins of the HTR-8/SVneo cells with different treatment times were obtained for subsequent detection of ACSL4 protein levels via Western blot.

### Co-Immunoprecipitation (Co-IP)

Co-IP assay was used to verify the binding between HMGB1 and ACSL4 in HTR-8/SVneo cells. The Co-IP complexes were purified by the PierceTM Crosslink Magnetic IP/Co-IP Kit (Thermo Fisher, 88805). The dosage of the IP antibody was 4 µg of anti-HMGB1 (Proteintech). The whole lysates and the antigen eluate were measured by Western blot.

### Statistical Analysis

Statistical analysis was performed using SPSS 22.0, GraphPad Prism 8 and Image J. Continuous variables in a normal distribution were expressed as mean ± SEM. Student’s t-test or Chi-square-test was used for comparison between two groups, ANOVA for comparison among three or more groups. Correlation was tested by Pearson’s correlation coefficient. Significant results were indicated by *P < 0.05 and **P < 0.01.

## Results

### HMGB1 and ACSL4 Expression Levels Increase in Villous Tissues from SA Women

**Fig. 1:**
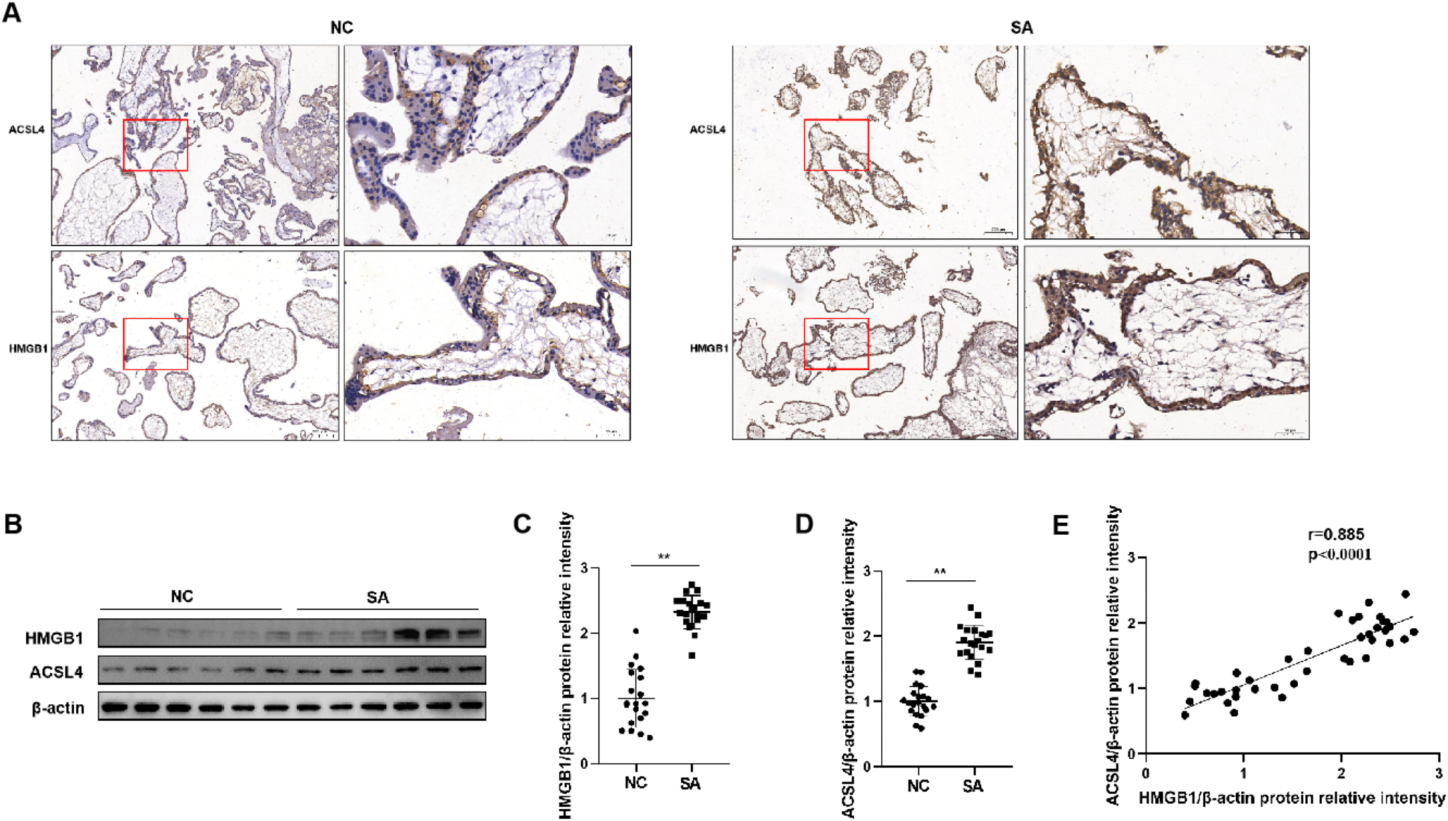
HMGB1 and ACSL4 expression in villous tissues from SA and normal control women. (**A**) The levels and location of HMGB1 and ACSL4 in villous tissues from SA and NC groups were measured by immunohistochemical staining. (**B**) The protein levels of HMGB1 and ACSL4 were determined by Western blot. **C** and **D**. Quantitative analysis of HMGB1 and ACSL4 in villous tissues from SA (*n* = 20) and NC (*n* = 20) groups, and the protein levels were normalized to β-actin. **E**. Correlation between HMGB1 and ACSL4 expression

Immunohistochemistry indicated that HMGB1 and ACSL4 were mainly expressed in the cytoplasm and partial nuclei of trophoblast cells, and their levels were higher in the villous tissues from patients with SA (Fig. 1A). The protein levels of both HMGB1 and ACSL4 increased in the SA group (Fig. 1B-D). Pearson’s correlation analysis revealed a significant positive correlation between HMGB1 and ASCL4 levels (Fig. 1E).

### Levels of Ferroptosis Hallmarks Increase in Villous Tissues from SA Women and LPS-treated HTR-8/SVneo Cells

**Fig. 2:**
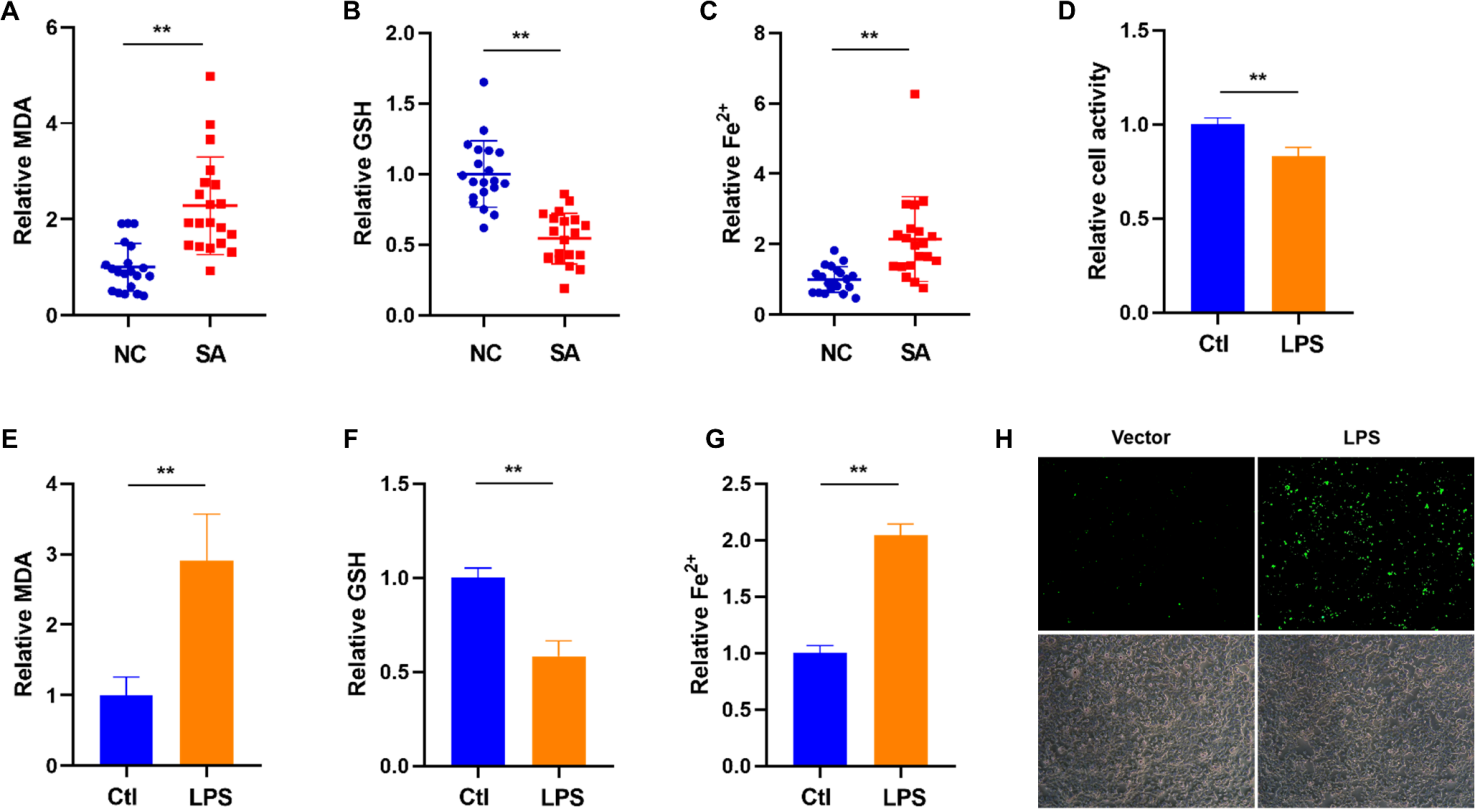
A higher level of ferroptosis in villous tissues from SA women and LPS-induced ferroptosis in HTR-8/SVneo cells. **A-C**. Analysis of MDA level (**A**), GSH content (**B**), and free Fe^2+^ ion level (**C**) in the SA (*n* = 20) and NC (*n* = 20) groups. **D-H**. Analysis of relative cell activity (**D**), MDA level (**E**), GSH content (**F**), and free Fe^2+^ ion level (**G**), ROS level (**H**) in LPS-induced HTR-8/SVneo cells

To examine whether ferroptosis is dysregulated in villous tissues from patients with SA and HTR-8/SVneo cells treated with LPS, we measured the levels of ferroptosis biomarkers, including MDA, GSH, Fe^2+^ and ROS. As expected, the SA group showed higher levels of MDA and Fe^2+^, and a lower level of GSH villous tissues than the NC group (Fig. 2A-C). Besides, LPS treatment significantly increased lipid peroxidation products, Fe^2+^ expression and ROS level, and decreased GSH expression (Fig. 2E-H).

### HMGB1 Promotes Ferroptosis in Trophoblasts

**Fig. 3:**
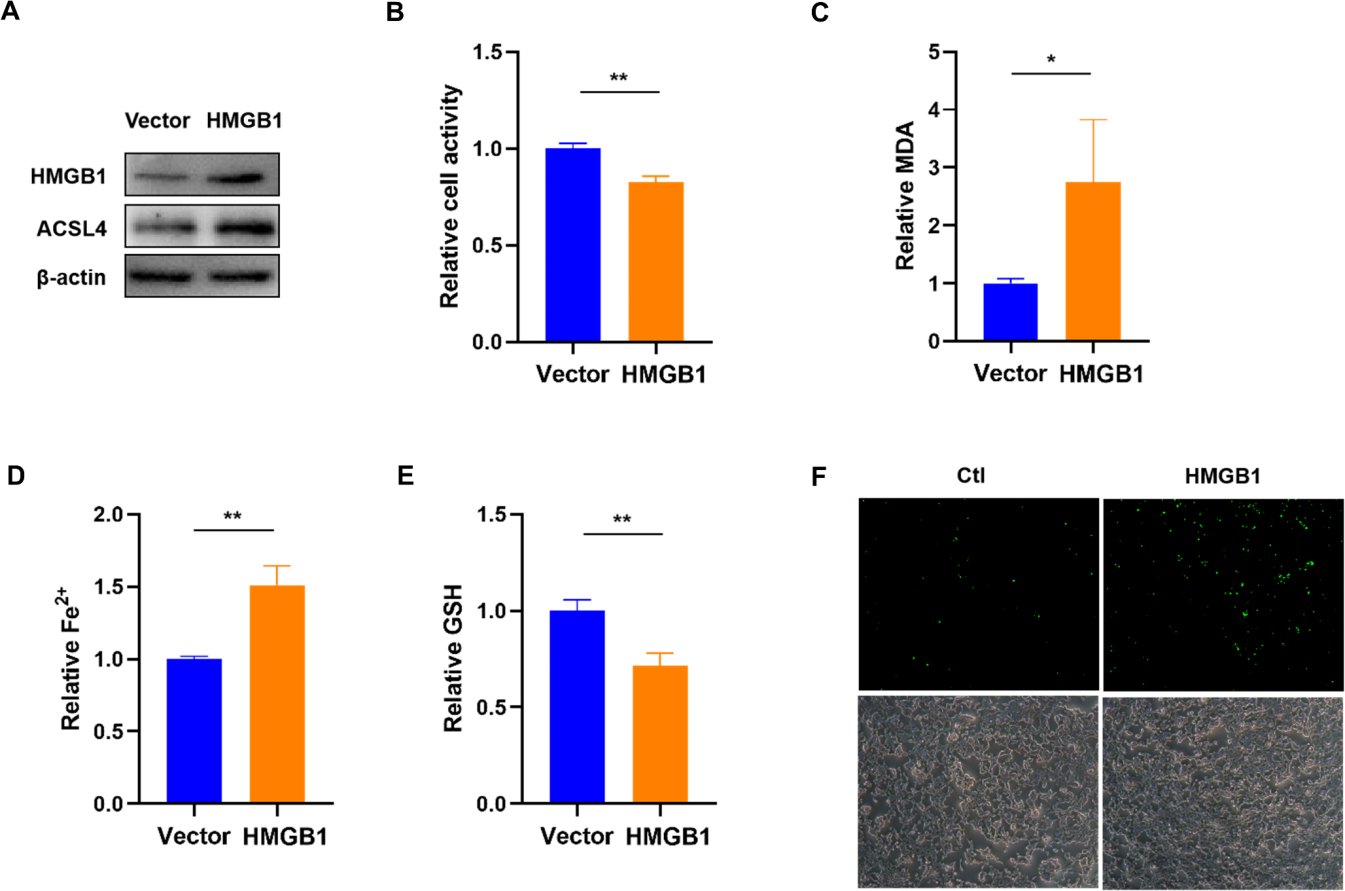
HMGB1 significantly promotes ferroptosis in HTR-8/SVneo cells. **A**. Western blot was used to measure HMGB1 and ACSL4 protein levels in HTR-8/SVneo cells treated with HMGB1 overexpression plasmids. **B-F**. Analysis of relative cell activity (**B**), MDA level (**C**), free Fe^2+^ ion level (**D**), GSH content (**E**), and ROS level (**F**) in HMGB1 overexpression HTR-8/SVneo cells

Western blot results demonstrated that HMGB1 overexpression plasmids increased HMGB1 protein level in HTR8/SVneo cells (Fig. 3A). As compared to the vector control group, HMGB1 overexpression markedly decreased the growth of HTR8/SVneo cells, as shown by the CCK-8 assay (Fig. 3B). As shown in Fig. 3C-E, we found that HMGB1 overexpression increased the levels of MDA and Fe^2+^, and inhibited the level of GSH. In addition, fluorescence showed that HMGB1 remarkably increased the level of ROS in HTR8/SVneo cells (Fig. 3F).

### ACSL4 Overexpression Enhances Ferroptosis in Trophoblasts

**Fig. 4:**
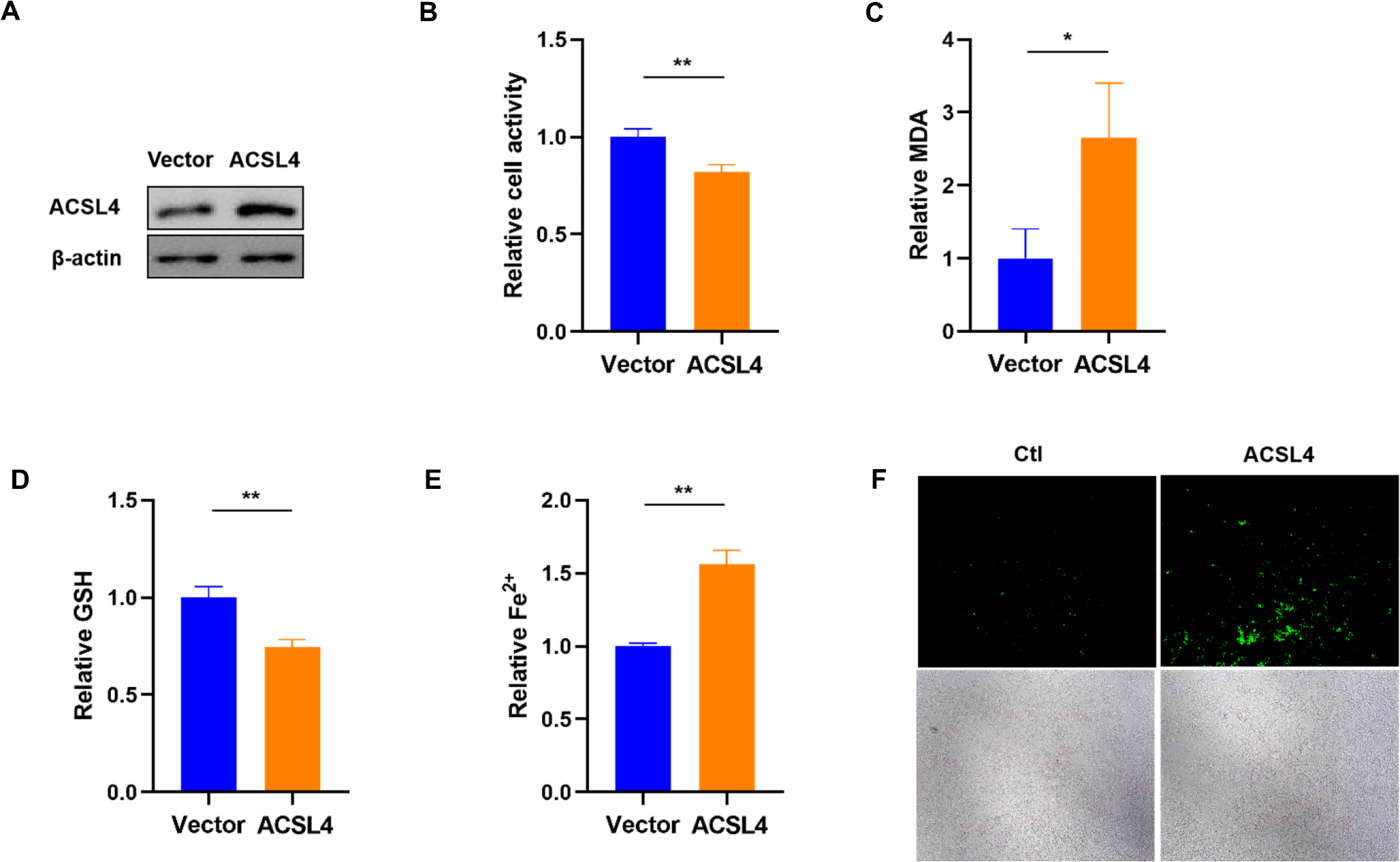
ACSL4 significantly promotes ferroptosis in HTR-8/SVneo cells. **A**. Western blot was used to measure ACSL4 protein level in HTR-8/SVneo cells treated with ACSL4 overexpression plasmids. **B-F**. Analysis of relative cell activity (**B**), MDA level (**C**), GSH content (**D**), and free Fe^2+^ ion level (**E**), ROS level (**F**) in ACSL4 overexpression HTR-8/SVneo cells

After confirming that ACSL4 overexpression plasmids could effectively upregulate ACSL4 (Fig. 4A), we examined the regulatory effects of ACSL4 on trophoblast cell growth and ferroptosis. The CCK-8 assay showed that ACSL4 overexpression in HTR8/SVneo cells inhibited cell growth (Fig. 4B). Furthermore, we observed that ACSL4 overexpression increased the levels of MDA, Fe^2+^ and ROS, and decreased GSH content (Fig. 4C-F).

### ACSL4 Silencing Attenuates HMGB1-induced Ferroptosis in Trophoblasts

**Fig. 5:**
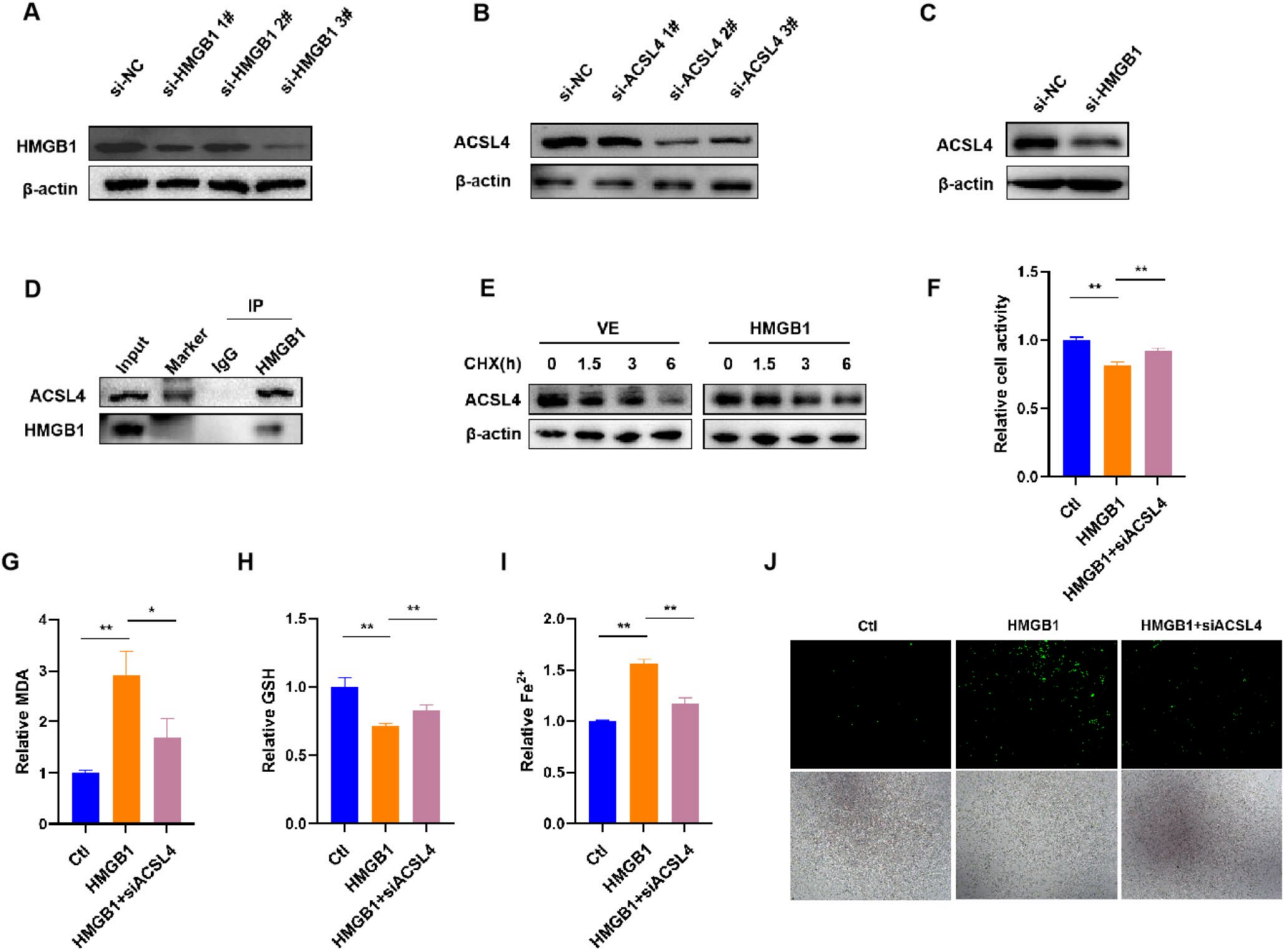
HMGB1 promotes trophoblastic ferroptosis by regulating ACSL4 expression. **A** and **B**. Western blot analysis of HMGB1 or ACSL4 protein levels in HTR-8/SVneo cells treated with HMGB1 or ACSL4 siRNA. **C**. Western blot analysis of ACSL4 protein level in HTR-8/SVneo cells treated with HMGB1 siRNA. **D**. ACSL4 conjugated with HMGB1 was detected by Co-IP assay. **E**. Effect of addition of 50 µM CHX to HTR-8/SVneo cells overexpressing HMGB1 and corresponding vector controls. Western blot is performed to detect changes in the half-life of the ACSL4 protein. **F-J**. HTR-8/SVneo cells treated with HMGB1 overexpression plasmids for 6 h were transfected with ACSL4 siRNA. Relative cell activity (**F**), MDA level (**G**), GSH content (**H**), and free Fe^2+^ ion level (**I**), ROS level (**J**) were analyzed

To confirm whether HMGB1 promotes ferroptosis in trophoblasts via upregulation of ACSL4 expression, first, we evaluated HMGB1 or ACSL4 expression in HTR8/SVneo cells treated with HMGB1 or ACSL4 siRNA. As shown in Fig. 5A, HMGB1 siRNA3# transfection markedly reduced HMGB1 protein level in HTR8/SVneo cells, and ACSL4 siRNA2# significantly inhibited ACSL4 protein expression (Fig. 5B). Then, HMGB1 siRNA3# and ACSL4 siRNA2# were subsequently selected for further study, showing that ACSL4 protein level increased in HMGB1-overexpressing cells (Fig. 3A) and decreased in HMGB1-deleted cells (Fig. 5C). What’s more, Western blot showed that ACSL4 was detected in the Co-IP products of HMGB1(Fig. 5D). The CHX chase assay subsequently confirmed that overexpression of HMGB1 proteins in HTR-8/SVneo cells attenuated the protein half-life of ACSL4 (Fig. 5E). We found that HMGB1 overexpression inhibited HTR8/SVneo cell growth, and this inhibition was then reversed by siRNA-mediated knockdown of ACSL4 (Fig. 5F). Similarly, downregulation of ACSL4 in HTR8/SVneo cells partly reversed the effects of HMGB1 on the levels of MDA, GSH, Fe^2+^ and ROS (Fig. 5G-J).

### LPS Promotes Ferroptosis via HMGB1/ACSL4 Pathway in Trophoblasts

**Fig. 6:**
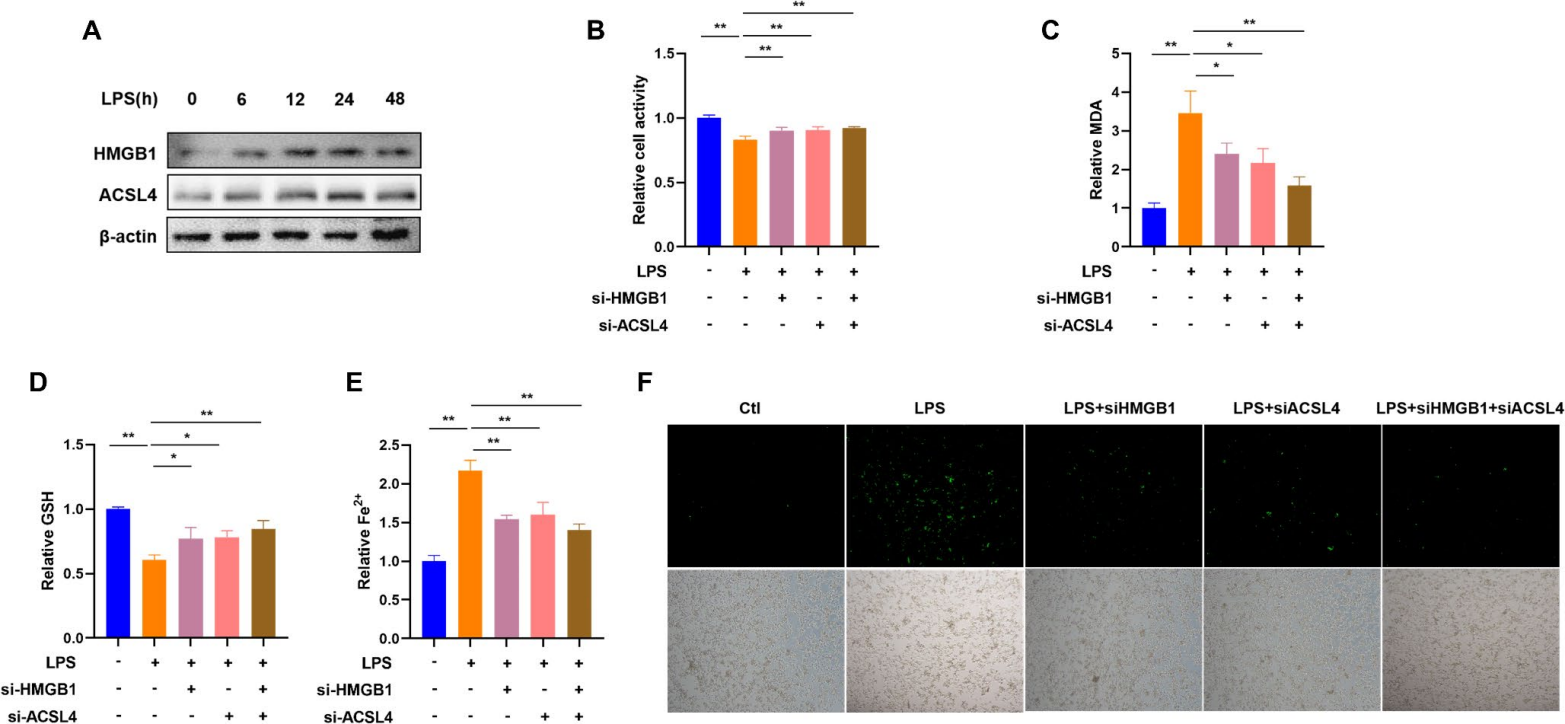
LPS promotes trophoblastic ferroptosis via the HMGB1/ACSL4 axis. **A**. Western blot analysis of HMGB1 and ACSL4 protein levels in HTR-8/SVneo cells treated with LPS for 0, 6, 12, 24, or 48 h. B-F. HTR-8/SVneo cells treated with LPS were transfected with HMGB1 or/and ACSL4 siRNA. Relative cell activity (**B**), MDA level (**C**), GSH content (**D**), and free Fe^2+^ ion level (**E**), ROS level (**F**) were analyzed

Western blot results showed that LPS notably raised HMGB1 and ACSL4 expression in HTR8/SVneo cells in a time-dependent manner (Fig. 6A). As shown in Fig. 6B, knockdown of HMGB1 or/and ACSL4 expression significantly promoted cell growth. A 24-h LPS treatment significantly increased the levels of MDA, Fe^2+^ and ROS, and decreased GSH content in HTR8/SVneo cells; however, HMGB1 or/and ACSL4 knockdown alleviated this effect (Fig. 6C-F).

## Discussion

This study showed that HMGB1 and ACSL4 were highly expressed in villous tissues from SA patients. LPS induced ferroptosis and increased HMGB1 and ACSL4 expression in trophoblasts. Inhibition on ACSL4 expression significantly suppressed HMGB1-induced ferroptosis, and inactivation of HMGB1/ACSL4 pathway reversed LPS-induced ferroptosis.

Abnormal ferroptosis leads to placental diseases, including pre-eclampsia, gestational diabetes mellitus, fetal growth restriction, and SA [6–8]. In SA, decidual stromal cells, endothelial cells, and trophoblast cells may all undergo excessive ferroptosis under stress. Abnormal iron metabolism, represented by iron overload, elicits ferroptosis in decidual stromal cells, thereby increasing lipid ROS and cell death [17]. As an organic pollutant and endocrine disrupting chemical, benzo(a)pyrene (BaP) and its metabolite BPDE enhance MARCHF1/GPX4-mediated ferroptosis, thus suppressing endothelial cell angiogenesis to cause miscarriage [18]. Cigarette smoke disrupts placental development in rats via inducing trophoblast cell ferroptosis [19]. Here, similar to previous studies [9, 20], we measured a high level of ferroptosis in trophoblast cells from SA women, which may be associated with overwhelming lipid peroxidation, iron overload, and excessive ROS.

LPS, as a initiator of inflammation, can be used to generate in vivo and in vitro models of various diseases, including neuroinflammatory, lung injury, and SA [1, 21, 22]. In this study, we also used LPS-induced HTR-8/SVneo cells to mimic SA in vitro. Although accumulated evidence suggests that the treatment with LPS causes acute inflammation in trophoblast cells, and abnormal levels of inflammatory cytokines in turn inhibit trophoblastic function. Here, we found that the levels of MDA, Fe^2+^ and ROS increased, and the content of GSH decreased in LPS-activated HTR-8/SVneo cells, validiting LPS as a potential inducer of ferroptosis. Given that LPS induces a significant increase in lipid peroxidation and ferroptosis in acute kidney injury [23], and ferroptosis is connected with inflammation [24], LPS may allow the cooperation between inflammation and ferroptosis, thus contributing to trophoblastic pathology and possible SA.

Increasing evidence has demonstrated that HMGB1, as a late-stage inflammatory agent, is involved in various inflammatory responses. Additionally, HMGB1 activates the release of inflammatory factors containing TNF-α and IL-1, which in turn increases the synthesis and release of HMGB1 [14]. HMGB1 is also essential for the development of SA. Xiaofeng Xu et al. have shown that the level of HMGB1 in the peripheral blood is higher in unexplained recurrent SA, compared with controls, and correlated with the level of IFN-γ, which can be blocked by the classic anti-inflammatory drug aspirin [15]. Similarly, here we reported that the level of HMGB1 was significantly higher in villous tissues from SA women than in controls. Feng Zhou et al. also mentioned that HMGB1 expression was mainly in cell nuclei of trophoblasts [1]. However, consistent with other study [25], our results showed that HMGB1 expression was also detected in cytoplasm, which participated in cell stress responses.

It is reported that HMGB1 promotes the inflammatory responses at the maternal-fetal interface of SA [1]. In addition, HMGB1 propels ferroptosis, and ferroptosis in turn promotes the pathophysiological procession of SA [16, 20]. On this basis, our data from trophoblasts with HMGB1 overexpression suggested that HMGB1 increased the levels of ferroptosis hallmarks. Our study also demonstrated that HMGB1 knockdown inhibited LPS-induced ferroptosis. Additionally, recent studies have shown that ferritinophagy and syringic acid regulate ferroptosis via HMGB1 [26, 27]. Beyond that, HMGB1 acts on many ferroptosis-related pathways. For example, Lili Guo et al. have demonstrated that SYVN1 attenuates ferroptosis via the HMGB1/NRF2/HO-1 axis to relieve spinal cord ischemia-reperfusion injury [28]. Glutathione S-transferase zeta 1 (GSTZ1) enhances ferroptosis through the HMGB1/GPX4 pathway in bladder cancer cells [29], indicating that HMGB1 promotes ferroptosis via inhibiting the pathways related to antioxidant defenses.

Our data showed that ACSL4 was abnormally overexpressed in villous tissues from SA women, which promoted trophoblastic ferroptosis. Recent studies have shown that ACSL4 facilitates the esterification of polyunsaturated fatty acids (PUFAs) to acyl-CoA containing AA (C20:4) and adrenic acid (C22:4) [30], both metabolites highly susceptible to oxidation induced by lipoxygenase, thereby initiating ferroptosis. The GPX4/ACSL4 axis mediates neuronal ferroptosis, pointing out that GPX4 may bridge the relationship between HMGB1 and ACSL4. Intriguingly, HMGB1 activates renal tubular ferroptosis through ACSL4 after ischemia/reperfusion injury [16]. Consistently, we demonstrated that HMGB1 mediated ACSL4-dependent trophoblastic ferroptosis induced by LPS via stabilizing ACSL4 expression, suggesting the critical role of an HMGB1/ACSL4 axis in SA.

The main limitation of our study is that we only used an in vitro model constructed with the human trophoblast cell line HTR8/SVneo. Our findings should be validated in in vivo studies in future.

In conclusion, HMGB1 promotes trophoblastic ferroptosis through stabilizing ACSL4 expression, and HMGB1 and ACSL4 inhibition reverses LPS-induced ferroptosis in HTR-8/SVneo cells. The HMGB1/ACSL4 pathway is involved in the trophoblast dysfunction related to SA, and may be exploited to design treatment options.

## Electronic Supplementary Material

Below is the link to the electronic supplementary material.

Supplementary Material 1

## Data Availability

The datasets used during the present study are available from the corresponding author upon reasonable request.
