# Supplementary material for: Increased Trophoblast Cell Ferroptosis via HMGB1/ACSL4 Pathway Is Associated with Spontaneous Abortion
Source: Reprod Sci. 2025 Feb 24;32(5):1713–22. doi: 10.1007/s43032-025-01817-7 (PMC12041038; doi:10.1007/s43032-025-01817-7)
Supplement: Supplementary file 1 — Supplementary Material 1 [file 43032_2025_1817_MOESM1_ESM.doc]

Dear editors,

We would like to submit the enclosed manuscript entitled “Increased trophoblast cell ferroptosis via HMGB1/ACSL4 pathway is associated with spontaneous abortion”, which we wish to be considered for possible publication in “***Reproductive Sciences***”. No conflict of interest exits in the submission of this manuscript, and manuscript is approved by all authors for publication. I would like to declare on behalf of my co-authors that the work described is an original research that has not been published previously, and not under consideration for publication elsewhere, in whole or in part.

Our study indicate that the levels of HMGB1 and ACSL4 in villous tissues from spontaneous abortion were significantly higher than the normal control group. HMGB1 promoted trophoblast cells ferroptosis through regulating ACSL4 expression. HMGB1 and/or ACSL4 inhibition attenuated LPS-induced trophoblast cells ferroptosis.

We deeply appreciate your consideration of our manuscript, and we look forward to receiving comments from the reviewers. If you have any queries, please don’t hesitate to contact me at the address below.

Thank you and best regards.

Sincerely yours,

Chengcai, Kong

Corresponding Author

Changzhou Maternal and Child Health Care Hospital, Changzhou Medical Center, Nanjing Medical University

16 Dingxiang Road, Changzhou 213000, China

[Tel:+86051988131510](tel:+86051988131510)

Fax: [+86](tel:+86)051988131510

E-mail: kongchengcai12@163.com
